# Paternal Nicotine/Ethanol/Caffeine Mixed Exposure Induces Offspring Rat Dysplasia and Its Potential “GC-IGF1” Programming Mechanism

**DOI:** 10.3390/ijms232315081

**Published:** 2022-12-01

**Authors:** Yi Liu, Cong Zhang, Yi Liu, Jiayong Zhu, Hui Qu, Siqi Zhou, Ming Chen, Dan Xu, Liaobin Chen, Hui Wang

**Affiliations:** 1 Department of Pharmacology, School of Basic Medical Sciences, Wuhan University, Wuhan 430071, China; 2 Department of Orthopedic Surgery, Zhongnan Hospital of Wuhan University, Wuhan 430071, China; 3 Hubei Provincial Key Laboratory of Developmentally Originated Disease, Wuhan 430071, China

**Keywords:** paternal nicotine/ethanol/caffeine mixed exposure, hypothalamic–pituitary–adrenal axis, reproductive function, adverse pregnancy outcomes, multiple organ dysfunction, offspring development

## Abstract

Clinical and animal studies suggest that paternal exposure to adverse environments (bad living habits and chronic stress, etc.) has profound impacts on offspring development; however, the mechanism of paternal disease has not been clarified. In this study, a meta-analysis was first performed to suggest that paternal exposure to nicotine, ethanol, or caffeine is a high-risk factor for adverse pregnancy outcomes. Next, we created a rat model of paternal nicotine/ethanol/caffeine mixed exposure (PME), whereby male Wistar rats were exposed to nicotine (0.1 mg/kg/d), ethanol (0.5 g/kg/d), and caffeine (7.5 mg/kg/d) for 8 weeks continuously, then mated with normal female rats to obtain a fetus (*n* = 12 for control group, *n* = 10 for PME group). Then, we analyzed the changes in paternal hypothalamic–pituitary–adrenal (HPA) axis activity, testicular function, pregnancy outcomes, fetal serum metabolic indicators, and multiple organ functions to explore the mechanism from the perspective of chronic stress. Our results demonstrated that PME led to enhanced paternal HPA axis activity, decreased sperm quality, and adverse pregnancy outcomes (stillbirth and absorption, decreased fetal weight and body length, and intrauterine growth retardation), abnormal fetal serum metabolic indicators (corticosterone, glucolipid metabolism, and sex hormones), and fetal multi-organ dysfunction (including hippocampus, adrenal, liver, ossification, and gonads). Furthermore, correlation analysis showed that the increased paternal corticosterone level was closely related to decreased sperm quality, adverse pregnancy outcomes, and abnormal offspring multi-organ function development. Among them, the decreased activity of the glucocorticoid-insulin-like growth factor 1 (GC-IGF1) axis may be the main mechanism of offspring development and multi-organ dysfunction caused by PME. This study explored the impact of common paternal lifestyle in daily life on offspring development, and proposed the GC-IGF1 programming mechanisms of paternal chronic stress-induced offspring dysplasia, which provides a novel insight for exploring the important role of paternal chronic stress in offspring development and guiding a healthy lifestyle for men.

## 1. Introduction

In recent years, developmentally derived diseases have become research hotspots worldwide. Previous studies have shown that maternal exposure to adverse environments during pregnancy can cause offspring dysplasia [1]. However, recent studies have shown that paternal factors also play an essential role in offspring development [2]. Cigarette smoking, alcohol, and coffee are the most common environmental agents in men’s daily habits. The National Health Interview Survey (NHIS) found that about 17.5% of men smoke, which means nicotine exposure, and most of them are 25–44 years old [3]. Ethanol is also known as alcohol, in the latest study, levels of alcohol consumption were classified as light, moderate, or heavy. Drinking more than five drinks or consuming more than 70 g of alcohol is considered heavy drinking [4]. As a type of xanthine alkaloid widely added to lots of beverages, caffeine is consumed by men at around 165 mg up to 230 mg per day in many countries, according to epidemiological research [5,6]. The impact of paternal exposure to cigarettes (nicotine), alcohol (ethanol), and coffee (caffeine) alone or in combination on the health of themselves and their offspring are becoming increasingly common. Animal studies have also indicated behavioral changes in rat offspring due to paternal nicotine exposure, manifesting as abnormal mobility and cognitive decline [7]. Paternal ethanol exposure can trigger intrauterine growth retardation (IUGR) in offspring, along with altered insulin signal and disrupted lipid homeostasis in the livers of offspring [8,9]. Considering the easy availability of nicotine, ethanol, and caffeine in daily life and its co-exposure [10,11], we proposed to investigate the effects of paternal mixed exposure (PME) with low doses of nicotine/ethanol/caffeine on offspring and their multi-organ development and its possible mechanisms.

Male reproductive function is evaluated with a focus on two parameters: androgen and sperm production. Male reproductive dysfunction has long been a global research hot topic. It has been reported that male infertility accounted for 20–30% per year among 48.5 million infertile couples in the world, which is closely related to men’s unhealthy lifestyle [12]. A clinical study with 2562 male subjects at Aarhus University Hospital in Denmark showed that male chronic smokers had 19% lower sperm concentrations and 29% lower sperm counts than non-smokers [13]. The linkage between alcohol and fertility was first investigated in 1985; studies found significant decreases on androgen levels, sperm volume, concentration, and motility in chronic alcoholics [14]. Moreover, the consumption of caffeinated beverages, such as cola, has been documented to have a negative impact on semen quality and prolonged pregnancy time [15]. Further, clinical evidence showed that the reproductive function of men with nicotine/ethanol mixed exposure was significantly lower than that of healthy men, along with an increased immature and abnormal sperm count [16,17]. These data suggested that PME could impair male reproductive function, yet its influences on offspring and potential mechanism haven not been reported.

Chronic stress refers to the nonspecific systemic reaction resulting in long-term stimuli of various internal and external factors [18]. Nicotine, ethanol, and caffeine are common paternal chronic stressors. Clinical and animal studies have confirmed that long-term exposure to nicotine, ethanol, or caffeine alone could activate paternal the hypothalamic–pituitary–adrenal (HPA) axis, accompanied by increased serum glucocorticoid (GC) concentration and a state of chronic stress [19,20,21,22]. Paternal chronic stress has been suggested to increase serum GC level and impair sperm epigenetic modification, ultimately leading to offspring dysplasia [23]. In addition, previous studies in our laboratory have found that prenatal exposure to nicotine, ethanol, or caffeine can activate the maternal HPA axis and bypass the placental GC barrier, resulting in fetal exposure to abnormal maternal GC levels and a changed glucocorticoid-insulin-like growth factor 1 (GC-IGF1) axis that eventually leads to IUGR occurrence, multi-organ development programming alterations, and susceptibility to multiple adult diseases [24,25,26]. Therefore, we could not help but speculate whether the PME model has a similar mechanism to the maternal nicotine, ethanol, or caffeine exposure model in that paternal high GC exposure causes abnormal offspring development by affecting sperm quality and whether GC-IGF1 axis programming is involved in this.

In this study, we first intended to determine whether paternal nicotine/ethanol/caffeine exposure was a risk factor for adverse pregnancy outcomes by using meta-analysis. Then, we constructed a rat PME model with low-dose nicotine (0.1 mg/kg/d), ethanol (0.5 g/kg/d), and caffeine (7.5 mg/kg/d) for 8 weeks continuously. The fetal rats were obtained by paternal inheritance, and we detected the HPA axis activity, testicular function, pregnancy outcome, fetal serum metabolic indicators, and multiple organ function gene expression. Finally, a series of correlation analyses were conducted to explore the possibility that PME causes multi-organ development abnormalities in offspring through GC-IGF1 axis programming. This study is helpful to guide men of childbearing age to live a healthy life and also provides a theoretical and experimental basis for the study of paternal origins of health and disease (POHaD).

## 2. Results

### 2.1. Meta-Analysis of the Population

**Figure 1:**
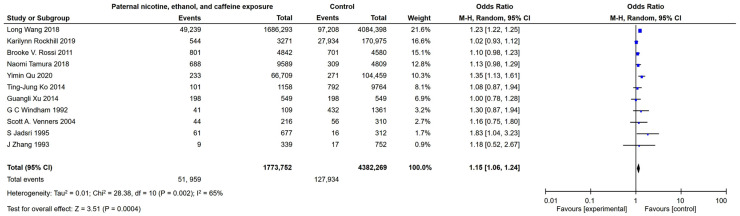
Meta analysis between paternal nicotine/ethanol/caffeine exposure and adverse pregnancy outcomes. Retrieval type: ((Male) OR (paternal) OR (father)) AND (((alcohol intake) OR (alcohol consumption) OR (alcohol drinking)) OR ((Caffeine intake) OR (Caffeine consumption) OR (coffee drinking)) OR ((Nicotine intake) OR (Nicotine consumption) OR (tobacco smoking))) AND ((spontaneous abortion) OR (miscarriage) OR (stillbirth) OR (preterm birth) OR (low birth weight) OR (small for gestational age infants) OR (Intrauterine Growth Retardation) OR (Growth Retardation, Intrauterine) OR (Intrauterine Growth Restriction) OR (Fetal Growth Restriction)). Study or subgroup [28,29,30,31,32,33,34,35,36,37,38].

Studies have suggested that paternal exposure to adverse environments can affect pregnancy [27]. In this meta-analysis, comprehensive literature retrieval has processed and covered all cases of control studies and cohort studies to provide evidence for the identification of the relationship between paternal exposure to nicotine, ethanol, and caffeine and adverse pregnancy outcomes (stillbirth, spontaneous abortion, premature birth, low birth weight, and intrauterine growth retardation) in population studies. We searched MEDLINE and the EMBASE database to find all studies published in any language as of 11 July 2021, to retrieve a total of 764 related articles. We filtered titles and abstracts to remove publications that were obviously irrelevant, such as editorials and individual case studies. Six members of the review team independently carried out the process of identifying relevant articles and extracting data; a total of 11 studies [28,29,30,31,32,33,34,35,36,37,38] (the PMID numbers were 29891638, 30615563, 21173655, 29466290, 31919145, 23850094, 24666755, 1510085, 15128612, 9037812, and 8427324, respectively) were included in the meta-analysis. The results showed that in the population analysis, the aggregate OR value for adverse pregnancy outcomes (including stillbirth, spontaneous abortion, premature delivery, low birth weight, and intrauterine growth retardation) under paternal exposure to nicotine, ethanol, and caffeine was 1.15 (95 % CI 1.06–1.24, *p* ≤ 0.01) (Figure 1). These results suggested that paternal mixed exposure (PME) to nicotine/ethanol/caffeine was a high-risk factor for adverse pregnancy outcomes.

### 2.2. PME Increases Paternal HPA Axis Activity and Impairs Testicular Function

**Figure 2:**
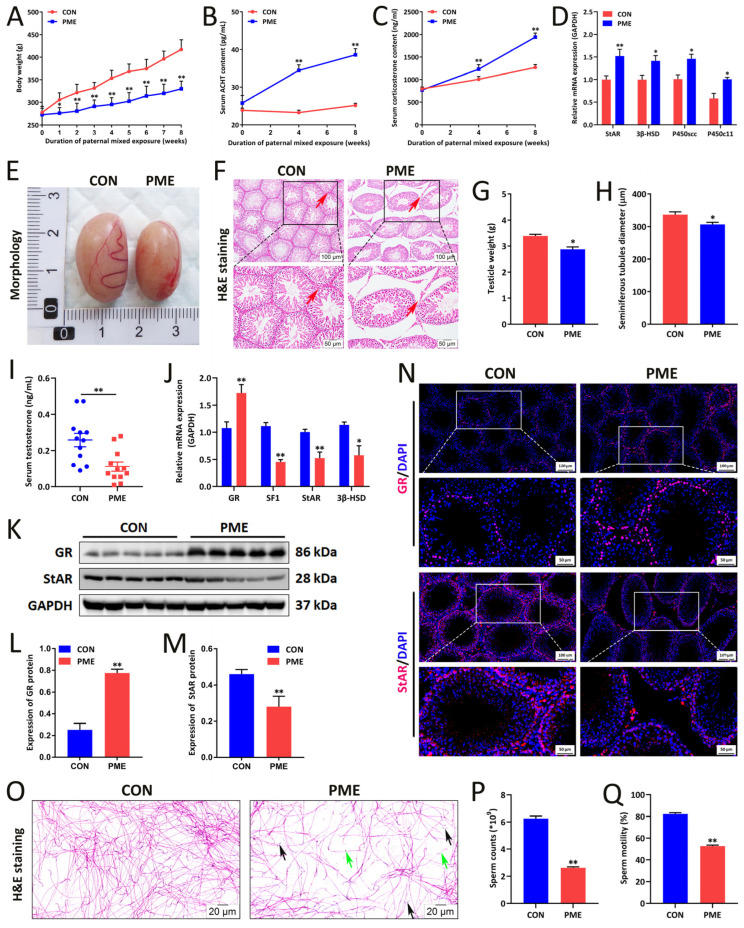
Paternal HPA axis function changes induced by nicotine/ethanol/caffeine exposure. (**A**) Body weight. (**B**) Serum adrenocorticotropic hormone (ACTH) content. (**C**) Serum corticosterone content. (**D**) StAR, 3β-HSD, P450c21, and P450c11 mRNA expression in paternal adrenal tissue. (**E**) Morphological appearance of the testis. (**F**) H&E staining of testis (the red arrows indicate pathological changes). Original magnification: 100× (scale bar: 100 μm); 200× (scale bar: 50 μm). (**G**) Testicular weight; (**H**) Testicular tube diameter; (**I**) Serum testosterone content. (**J**) StAR, 3β-HSD, and P450scc mRNA expression in testis. (**K**–**M**) Representative immunoblot images of GR and StAR protein expression in testis (**K**) and densitometric analysis (**L**,**M**). (**N**) Representative images of immunofluorescence assay of GR and StAR in testis sections. Original magnification: 100× (scale bar: 100 μm); 200× (scale bar: 50 μm). (**O**) H&E staining of sperm (green arrow: headless sperm; black arrow: tailless sperm). Original magnification: 400×; scale bar: 20 μm. (**P**) Sperm counts. (**Q**) Sperm mobility. Means ± S.E.M, * *p* < 0.05, ** *p* < 0.01 vs. CON (control), *n* = 10 for RT-qPCR analysis and serum biochemical detection; *n* = 5 for Western bolt analysis.

Studies have shown that nicotine, ethanol, and caffeine are the neuroexcitatory substances that can activate the HPA axis and impair testicular function [10,39]. Therefore, we constructed a rat model of mixed nicotine/ethanol/caffeine exposure to investigate its impact on paternal reproductive function and offspring development. First, we measured the paternal body weight and HPA axis activity before and after administration. The results showed that with the longer dosing time and compared with the control group, the weight and weight growth rate of rats in the PME group gradually decreased (Figure 2A) and serum adrenocorticotropin (ACTH) and corticosterone levels were gradually increased (Figure 2B,C); however, there was no significant changes in serum glucose and lipid indicators (Figure S1). The RT-qPCR analysis demonstrated that the mRNA expression of genes involved in the adrenal steroidal synthase system (StAR, P450scc, 3β-HSD, P450c21, and P450c11) were significantly increased after parental administration (Figure 2D). These results suggested that PME could inhibit paternal weight gain and increase HPA axis activity.

**Figure 3:**
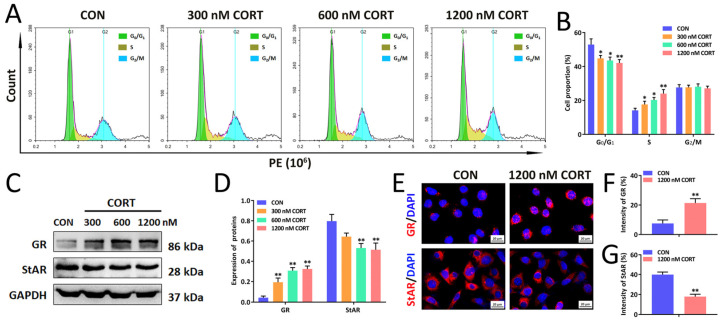
Corticosterone treatment suppresses GC-1 cell proliferation and decreases StAR expression. (**A**,**B**) Flow chart of cell cycle distribution (**A**) and cell cycle percentage statistics (**B**). (**C**,**D**) Representative immunoblot images of GR and StAR protein expression in GC-1 cells (**C**) and densitometric analysis (**D**). (**E**–**G**) Representative images of immunofluorescence assay of GR and StAR in GC-1 cells (**E**) and densitometric analysis (**F**,**G**). Original magnification: 400×, scale bar: 20 μm. Means ± S.E.M, *n* = 5, * *p* < 0.05, ** *p* < 0.01 vs. CON (control).

Further, we investigated the impact of mixed nicotine/ethanol/caffeine exposure on testicular morphology and testosterone synthesis and spermatogenesis function in fathers [40]. The results showed that the paternal testicular volume (Figure 2E) and weight (Figure 2G) in the PME group were significantly reduced in comparison with the control group. Pathologically, H&E staining of testis demonstrated widened interstitial area, decreased Leydig cell numbers, disordered arrangement of Sertoli cells (red arrows) (Figure 2F), and shortened spermatogenic tubule diameter (Figure 2H). Meanwhile, the testicular expression of key markers for cell proliferation, Ki67 and PCNA, were inhibited in the PME group (Figure S2). Moreover, the level of serum testosterone was decreased (Figure 2I) and the mRNA levels of testicular GR were increased along with decreased levels of key genes of the testosterone synthetase system (StAR and 3β-HSD) (Figure 2J). Western blotting and immunofluorescence results also demonstrated that PME increased the testicular protein expression of GR but decreased the protein expression of StAR (Figure 2K–N). In addition, the sperm motility video and H&E staining results showed slower sperm swam speed, shorter swam distance, lower motility (Supplementary Videos S1 and S2), and higher abnormal sperm count (headless and tailless, etc.) in the PME group compared with the control group (Figure 2O), and both sperm counts and motility were significantly decreased (Figure 2P,Q). Furthermore, we investigated the impact of corticosterone in spermatogonia GC-1 proliferation viability and StAR expression. The results indicated that different concentrations of corticosterone (CORT) (300–1200 nM) suppressed GC-1 cell proliferation viability and arrested GC-1 cell cycle in S phase (Figure 3A,B). There was also decreased StAR expression (Figure 3C–E). In conclusion, PME could lead to testicular morphologic changes, testosterone synthesis inhibition, and abnormal spermatogenesis.

### 2.3. Influences of PME on Pregnancy Outcomes and Offspring Development

**Figure 4:**
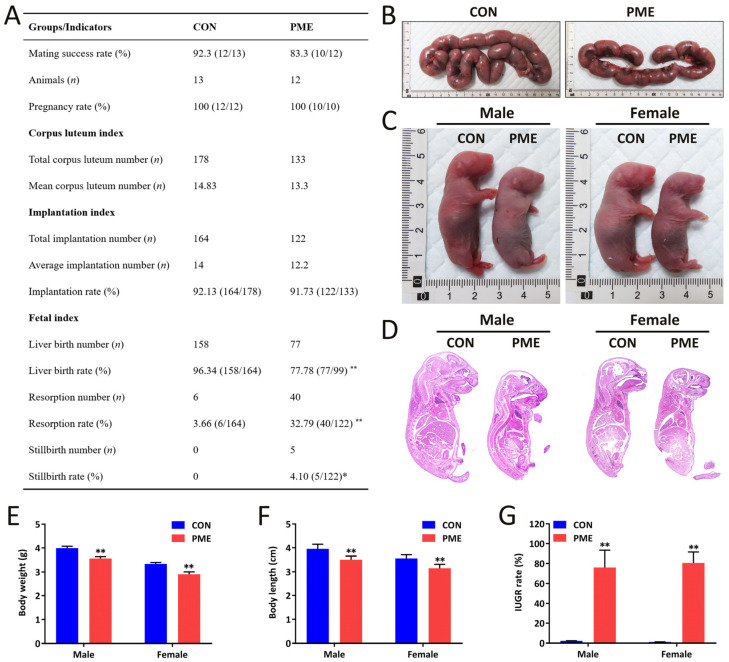
Adverse pregnancy outcomes induced by paternal mixed exposure (PME) to nicotine/ethanol/caffeine. (**A**) Pregnancy-outcome-related indicators. (**B**) Morphology of the fetus. (**C**) Morphology of male and female fetal rats. (**D**) Whole-body H&E staining of male and female fetal rats. Original magnification: 4×, scale bar: 200 μm. (**E**) Fetal body weight. (**F**) Fetal body length. (**G**) IUGR rate. Means ± S.E.M., * *p* < 0.05, ** *p* < 0.01 vs. CON (control).

In order to further clarify the influence of PME on pregnancy outcomes and offspring development, we observed the changes in paternal fertility and pregnancy outcomes. Compared with the control group, the results showed no significant changes in paternal mating success rate, pregnancy rate, average luteal number, and implantation rate in the PME group (Figure 4A,B). However, the live birth rate was significantly decreased (Figure 4A), whereas the rates of stillbirth and absorption were significantly increased (Figure 4A). The morphological and H&E analysis of whole body in fetal rats showed that the size, body weight, and length of male and female fetus was reduced (Figure 4C–F) and the IUGR rate was significantly enhanced (Figure 4G). All these results suggested that PME could impair pregnancy outcomes and offspring development, mainly manifesting as the decreased live birth rate, increased stillbirth and absorption rates, decreased fetal body weight and length, and increased IUGR rate.

### 2.4. Impairments of PME on HPA Axis Activity, Glucolipid Metabolism, and Sex Hormone Levels in Offspring

**Figure 5:**
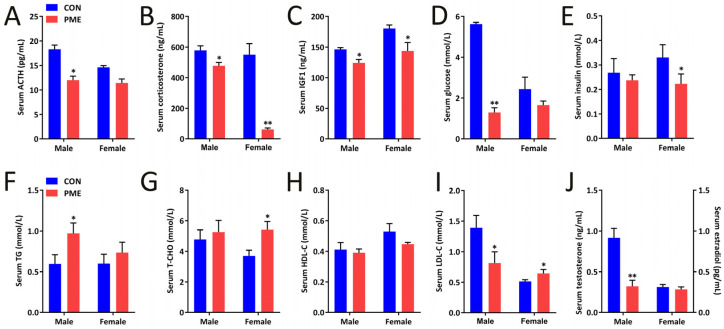
Impairments of paternal mixed exposure (PME) on HPA axis activity, glucolipid metabolism, and sex hormone levels in offspring. (**A**) Serum ACTH content. (**B**) Serum corticosterone content. (**C**) Serum IGF1 content. (**D**–**I**) Levels of serum glucolipid metabolism indicators, including glucose (**D**), insulin (**E**), TG (**F**), T-CHO (**G**), HDL-c (**H**), and LDL-c (**I**). (**J**) Serum testosterone and estradiol contents. Means ± S.E.M., *n* = 10 * *p* < 0.05, ** *p* < 0.01 vs. CON (control).

**Figure 6:**
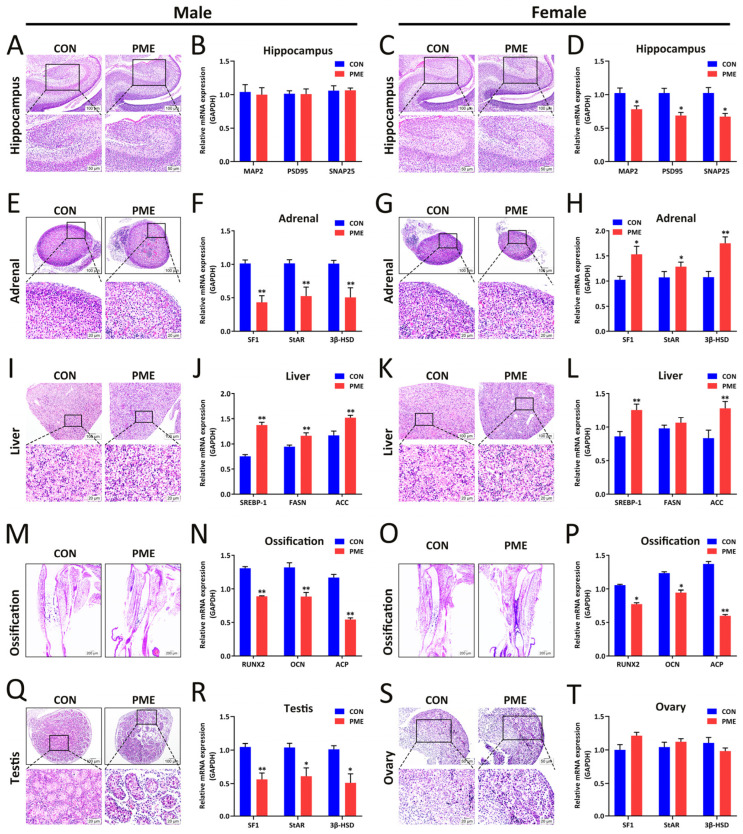
Paternal mixed exposure (PME) to nicotine/ethanol/caffeine induces multiple organ dysfunction in offspring. (**A**,**C**) H&E staining of the hippocampus in male and female fetuses. (**B**,**D**) mRNA expression of MAP2, PSD95, and SNAP25 in male and female fetal hippocampi. (**E**,**G**) H&E staining of the adrenals in male and female fetuses. (**F**,**H**) mRNA expression of SF1, StAR, and 3β-HSD in male and female fetal adrenals. (**I**,**K**) H&E staining of the livers in male and female fetuses. (**J**,**L**) mRNA expression of SREBP-1, FASN, and ACC in male and female fetal livers. (**M**,**O**) H&E staining of the ossifications in male and female fetuses. (**N**,**P**) mRNA expression of RUNX2, DCN, and ACP in male and female fetal ossifications. (**Q**,**S**) H&E staining of the testis (**Q**) and ovary (**S**) in fetuses. (**R**,**T**) mRNA expression of SF1, StAR, and 3β-HSD in fetal testis (**R**) and ovaries (**T**). Means ± S.E.M, *n* = 10, * *p* < 0.05, ** *p* < 0.01 vs. CON (control).

Furthermore, we detected a series of fetal serum metabolic indicators, including HPA axis activity, glycolipid metabolic indexes, and sex hormone levels. In male offspring, the serum ACTH, corticosterone, insulin-like growth factor 1 (IGF1), glucose, LDL-c, and testosterone levels were significantly decreased (Figure 5A–D and Figure 6I,J), with increased serum TG content (Figure 5F), but no significant changes in the serum insulin, T-CHO, and HDL-c levels (Figure 5E,H,I) compared with the control group. In female offspring, the serum corticosterone, IGF1, and insulin levels were significantly decreased (Figure 5B,C,E) and serum T-CHO and LDL-c levels were significantly increased (Figure 5F,G), but no significant changes in serum ACTH, glucose, TG, HDL-c, and estradiol levels (Figure 5A,D,E,H,J). Furthermore, serum corticosterone levels in male and female fetal serum were positively correlated with the body weight, body length, and serum IGF1 level in both control and PME groups and the correlations in the PME group were better than in the control group (Table 1). These results suggest that PME could cause a variety of serum metabolic indicators changes in the male and female fetal rats, including HPA axis activity, sex hormone level, and glycose and lipid metabolism, and that there are some gender differences.

### 2.5. PME Induces Multiple Organ Dysfunction in Offspring

Next, we looked for functional impairment due to PME in multiple organs in offspring, including hippocampal synaptic development [41], adrenal steroid synthesis function, hepatic lipogenesis [42], osteogenic function [43], and testicular/ovarian sex hormone synthesis [44]. The results showed that there were no significant differences on hippocampal morphology in both male and female fetus (Figure 6A,C) of the PME group compared with that of the control group, and no changes in the mRNA expression of MAP2, PSD96, and SNAP26 in the hippocampus of male PME fetuses (Figure 6B), along with decreased levels of MAP2, PSD96, and SNAP26 in female PME fetuses (Figure 6D). In addition, the maximum cross-sectional areas of the adrenal gland were decreased in male and female PME fetuses (Figure 6E,G) and the SF1, StAR, and 3β-HSD mRNA levels of the adrenal gland were significantly decreased in male PME fetuses (Figure 6F) but increased in female PME fetuses (Figure 6H). Moreover, there was obvious steatosis in the livers of male and female PME fetuses and the mRNA levels of SREBP-1, FASN, and ACC in the liver were significantly increased (Figure 6I–L). Furthermore, the mRNA levels of osteogenic RUNX2, OCN, and ACP in ossification were significantly decreased in male and female PME fetuses (Figure 6M–P), along with a shorter tibial length. Meanwhile, the testicular interstitium was wider, interstitial cell arrangement was disordered, and the mRNA levels of SF1, StAR, and 3β-HSD in the testis were significantly decreased (Figure 6Q,R), whereas the morphology of the ovaries in female PME fetuses and the mRNA levels of SF1, StAR, and 3β-HSD had no obvious changes (Figure 6S,T). These findings indicated that PME could not only enhance lipogenesis in the livers and inhibit osteogenic differentiation in both male and female offspring but also, with obvious gender differences, alter multi-organ morphology and functions, exhibiting as adrenal gland and testicular function inhibition in male offspring and hippocampal function suppression and hippocampal function enhancement in female PME offspring.

### 2.6. Paternal HPA Axis Activity Is Associated with Testicular Reproductive Function, Pregnancy Outcome, and Offspring Development

**Figure 7:**
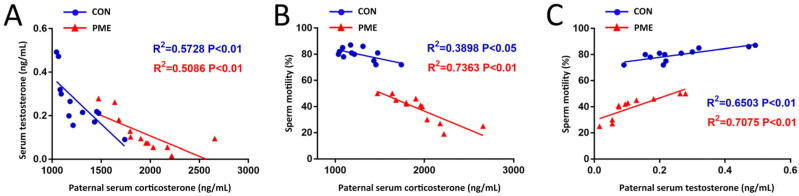
Paternal correlation analyses among serum corticosterone, testosterone levels, and sperm motility in paternal caffeine/nicotine/ethanol exposure rats. (**A**) Correlation between paternal serum corticosterone and testosterone. (**B**) Correlation between paternal serum corticosterone and sperm motility. (**C**) Correlation between paternal serum testosterone and sperm motility. PME, paternal mixed exposure.

To further explore the internal relationship among paternal HPA axis activity, testicular reproductive function, and offspring multi-organ developmental toxicities in the PME group, we conducted a series of correlation analyses among these indicators. First, we analyzed the correlations among paternal HPA axis activity (serum corticosterone levels) and testicular function (serum testosterone levels and sperm motility). The results showed that serum corticosterone levels were negatively correlated with serum testosterone levels and sperm motility in the control and PME groups (Figure 7A,B), whereas serum testosterone levels were positively correlated with sperm motility (Figure 7C). Among them, the correlation (R^2^ = 0.7363) between serum corticosterone level and sperm motility was the highest in the PME group. These results suggest that PME may lead to decreased sperm motility, mainly through paternal GC overexposure.

In order to further confirm that PME may affect pregnancy outcome and offspring multi-organ development through “high blood corticosterone—low sperm motility”, we conducted a series of correlation analyses among paternal and fetal rat relative indicators. The results showed that there was no correlation among paternal serum corticosterone and fetal physical development/serum phenotype in the control group (Table S1). However, in the PME male fetal fetuses, there were significant negative or positive correlations among paternal indexes (serum corticosterone level and sperm motility) and fetal physical development indexes (e.g., body weight and length) or multi-organ function indexes (e.g., serum corticosterone, IGF1, and testosterone levels) (Table 2). Meanwhile, in the female PME fetuses, paternal indexes (serum corticosterone level and sperm motility) were significantly negatively or positively correlated with fetal body weight or serum corticosterone level and paternal sperm motility was significantly positively correlated with fetal serum IGF1 level (Table 2). However, paternal indicators did not correlate with other phenotypic indicators of fetal serum, including glucose and lipid metabolism and estrogen level (Table 2). These results suggest that paternal GC overexposure in the PME group leads to abnormal male and female fetal multi-organ development through low sperm motility.

## 3. Discussion

### 3.1. Basis for PME Rat Model Establishment and Its Toxicological Significance

Nicotine, ethanol, and caffeine are the main components of tobacco, alcoholic products, and beverages, respectively, that are commonly consumed in daily lives. Our study first confirmed that paternal consumption of tobacco, alcohol, and caffeine was a high risk factor for adverse pregnancy through a meta-analysis of cohort data. Male chronic smokers smoke about 20 cigarettes or more per day with a nicotine intake of about 3.163 mg/day [45,46]. Male alcoholics consume about 180 mL of alcoholic products daily with 40–50% alcohol content [19]. The average caffeine intake in American men is 165 mg/day, with a maximum of 230 mg/day [5]. Combining these data, an adult man weighing 70 kg would have a nicotine intake at 0.045 mg/kg/d, ethanol at 1.5 g/kg/d, and caffeine at 2.36 mg/kg/d. An animal model with paternal mixed exposure (PME) to nicotine/ethanol/caffeine was established herein. In detail, nicotine, alcohol, and caffeine were given to male rats at PW8 (sexual maturity to produce sperm) and continued for 8 weeks, a complete spermatogenesis cycle [47]. In terms of the dose, each male rat was administered nicotine 0.1 mg/kg/d, ethanol 0.5 g/kg/d, caffeine 7.5 mg/kg/d, which matches 0.016 mg/kg/d nicotine, 0.083 g/kg/d ethanol, and 1.21 mg/kg/d caffeine in a human based on a human–rat dose conversion (1:6.17), to make sure that the exposure dose of each exogenous substance was lower than the daily intake [48,49]. In conclusion, this study developed a rat model of PME to low-dose nicotine/ethanol/caffeine based on their daily intakes in men and spermatogenic features, followed by investigating the effects of PME on paternal HPA axis activity, reproductive function, and fetal development (including serum metabolic indicators and multi-organ function), to explore the possible chronic stress (GC) programming mechanisms.

### 3.2. PME May Impair Paternal Reproductive Function by Activating the HPA Axis

It is known that testicular function mainly comprises testosterone synthesis and spermatogenesis, which are susceptible to exogenous factors [50]. Androgen plays an important role in maintaining male reproductive function such as spermatogenesis, sperm count, and motility. Studies have found that male smokers and alcoholics have insufficient testicular androgen production and sperm quality [13]. In addition, the abnormal paternal reproductive function can also affect offspring development, as demonstrated in that overexposure to dioxins contributes to the increased incidence of abnormal sperm morphology and adverse pregnancy outcomes [51,52]. Recent studies have also found that various paternal factors (such as irregular work and rest, bad eating habits, abnormal environmental exposure, etc.) can lead to adverse pregnancy outcomes and even affect offspring health [53,54]. In this study, we found that male rats in the PME group manifested testicular morphology changes (reduced volume, widened interstitial area, and shortened area/diameter of spermatogenic tubules), inhibited testosterone synthesis function (decreased serum testosterone level and reduced testicular StAR/P450scc expression), and abnormal spermatogenesis (decreased sperm count/motility and increased abnormal sperm count). Correlation analysis showed that serum testosterone level was significantly positively associated with sperm motility. Furthermore, although PME had no significant impact on paternal fertility and maternal conception, it could impair pregnancy outcomes and fetal multi-organ development, mainly manifested as the decreased live-birth rate and the increased stillbirth and absorption rates, accompanied by decreased fetal body weight and length and increased IUGR rate. These results strongly suggested that PME might contribute to the decreased sperm motility and adverse pregnancy outcomes by inhibiting testosterone synthesis.

Chronic stress refers to the nonspecific systemic reaction caused by the long-term stimuli of various internal and external factors [55], such as paternal psychological stress and long-term depression [56]. Various physiological functions will be changed under long-term chronic stress, among which elevated serum glucocorticoid level is the most typical [57]. Nicotine, ethanol, and caffeine all have a specific nerve excitatory effect [58]. Human and animal studies have shown that male chronic smokers, alcoholics, and coffee drinkers showed a level of excitement on the HPA axis, and elevated serum ACTH and cortisol levels, an apparent chronic stress state [19,20,59]. Reproductive function inhibition of male rats under long-term chronic stress is associated with increased serum corticosterone levels [60,61]. Our previous study found that prenatal exposure to nicotine, ethanol, or caffeine induced multi-organ dysplasia in offspring mainly attributed to an activated maternal HPA axis [62,63,64]. In this study, we found that the body weight and growth rate of paternal rats in the PME group were gradually decreased in a time-dependent manner but the activity of the HPA axis and the function of adrenal steroid synthesis were significantly increased. These results suggested that PME could induce paternal chronic stress, mainly presented as elevated HPA axis activity. Correlation analysis also showed that that elevated levels of serum ACTH and corticosterone were significantly negatively correlated with reduced serum testosterone level and sperm motility. These findings suggested that PME might lead to decreased sperm motility and adverse pregnancy outcomes, mainly through paternal high GC exposure.

### 3.3. PME Can Cause Fetal Multi-Organ Dysplasia with Obvious Organ Specificity and Sex Differences

The intrauterine period is known to have high susceptibility in development, and the fetus can be easily affected by multiple factors such as sperm–egg fusion and the intrauterine environment [65]. Studies have shown that paternal chronic stress could induce multiple organ dysfunction in offspring, such as increased anxiety and depression-like behavior and changes in glucose and lipid metabolism [66,67]. Our previous studies have confirmed that prenatal exposure to nicotine, ethanol, or caffeine could cause low birth weight, high IUGR rate, and multi-organ dysfunction mainly through maternal glucocorticoid overexposure and enhanced susceptibility to adult diseases [26,62,68].

In this study, we found that PME could induce a variety of serum metabolic indicators in fetal rats, including HPA axis activity, sex hormone level, and glucolipid metabolism. In addition, PME could cause abnormal expression of multi-organ functional genes in fetal rats. Among them, both male and female fetal rats of the PME group showed increased hepatic lipid synthesis and inhibited osteogenesis differentiation, which also existed in the other models of adverse environmental exposure during pregnancy [68,69]. Considering that the liver is the main metabolic organ in the fetal period, which is very crucial for fetal development [70], and bone is a non-critical organ in utero that develops throughout the fetal period [71], these two organs may be more sensitive to an adverse external environment and more prone to show pathological changes when affected by adverse external factors that may be explained by the “developmental plasticity” and “thrifty phenotype” [26], because they need to adapt to the altered intrauterine environments caused by PME. Meanwhile, we found that the HPA axis activity and sex hormone levels were significantly reduced in the male PME fetuses, whereas the glucolipid metabolic phenotype was abnormal in the females with a significant gender difference. The adrenal steroid synthesis was obviously inhibited in the male PME offspring but enhanced in the females. Such sex differences in adrenal function have been reported in postnatal offspring with dexamethasone exposure during pregnancy [72]. In addition, testis testosterone synthesis is significantly inhibited in the male PME offspring, whereas ovary estrogen synthesis has no significant change in the females. All these results suggest that the gender differences in multi-organ function is a common phenomenon. In conclusion, PME can cause a variety of fetal serum metabolic indicators and multi-organ function changes in offspring, with obvious organ specificity and gender differences.

### 3.4. “GC-IGF1” Axis Programming Alteration Might Be Involved in Fetal Multi-Organ Dysplasia by PME

It is known that IGF1 is a kind of local factor with extensive effects. Blood IGF1 mainly comes from the fetal liver during the intrauterine period and is involved in cell proliferation, differentiation, and metabolism in various fetal tissues. Autocrine or paracrine IGF1 gradually appears in various organs in late pregnancy [73]. It has been reported that IGF1 is involved in the regulation of fetal physical and multi-organ development, such as adrenal steroid synthesis function [73], gonadal sex hormone synthesis [74], and bone development [75]. In this study, PME could significantly reduce serum IGF1 level in both female and male fetal rats, which could explain why both PME female and male fetal rats showed reduced body weight and body length. At the same time, the functional development of multiple organs of male fetal rats in the PME group was inhibited, including the hippocampus, adrenal gland, gonad, bone, etc., which is also seemed to be explained by the decreased serum IGF1 level. However, in female PME fetal rats, some serum metabolic indicators and multi-organ functions did not change significantly (such as serum estrogen, and TCH, LDL-C). This gender difference may be related to the susceptibility of female fetal development to maternal hormones (such as GC and estrogen) during the intrauterine period, including the placental glucocorticoid barrier [76], placental nutrient transport function [77], and maternal estrogen level [78]. It is a common phenomenon that maternal factors lead to less obvious intrauterine changes in female fetuses than in male fetuses, which may be related to the protective effect of the mother on her female fetuses.

The HPA axis is known to be involved in regulating stress responses and circadian rhythms, and the adrenal gland is one of the fastest and earliest developing organs in the HPA axis [68]. Intrauterine serum GC is mainly derived from the mother and gradually increases with the progress of pregnancy [73]. Studies have found that there is a positive correlation between serum GC and IGFI levels during fetal physiological development [79]. These results suggest that the “GC-IGF1” axis positively regulates fetal physiological development. In addition, a series of recent studies in our laboratory have found that dexamethasone exposure during pregnancy can inhibit maternal adrenal function, resulting in the reduction of maternal and fetal serum corticosterone levels, whereas fetal serum GC with low physiological concentrations can reduce the development of the adrenal glands, testis, and long bones of offspring through positive programming of the “GC-IGF1” axis [73,74,75]. These results suggest that the positive programming of the GC-IGF1 axis is also involved in fetal development under pathological states. In this study, we found that among the various fetal serum metabolic indicators induced by PME, the changes in serum hormone levels were significantly greater than those in serum glucolipid metabolism. We further found that the serum corticosterone and IGF1 levels were decreased in the female and male PME fetal rats and that the correlation between them was better than that in the control group. These results suggest that there may be low functional programming of the “GC-IGF1” axis in the PME fetal rats, leading to multi-organ dysplasia. Interestingly, our further correlation analysis showed that there was a significant negative correlation between the serum corticosterone level in parental rats and the serum corticosterone and IGF1 levels in female and male fetal rats. Combined with domestic and foreign studies and our previous conclusion of “maternal intrauterine GC overexposure inhibits fetal development” [62,65,68], it is suggested that the positive change in serum “GC-IGF1” in the PME fetuses is related to the low sperm motility caused by paternal GC overexposure leading to the inhibition of fetal development. However, this is worthy of further investigation.

### 3.5. Summary

**Figure 8:**
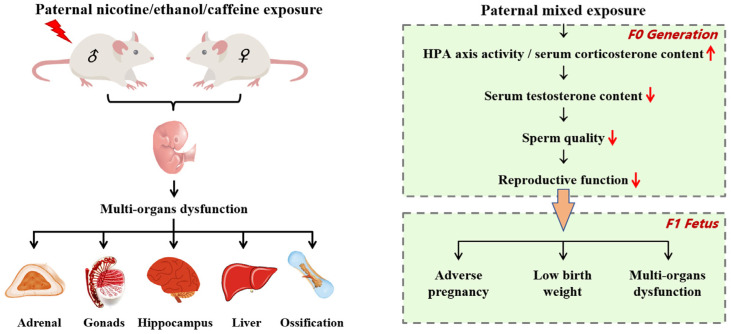
Paternal nicotine/ethanol/caffeine mixed exposure induces adverse pregnancy outcomes and offspring multi-organ dysfunction. ↑: elevate; ↓: decrease.

In this study, we used population meta-analysis to reveal PME as a key risk factor for adverse pregnancy and established a rat PME model for the first time by simulating unhealthy lifestyles in humans to confirm that PME to nicotine/ethanol/caffeine can lead to fetal dysplasia with gender differences that may be related to the activation of the HPA axis by PME leading to paternal reproductive dysfunction and adverse pregnancy outcomes and the programming of “GC-IGF1” axis leading to fetal multiple organ dysfunction (Figure 8). The current study provides guidance for men of childbearing age to live a healthy life and provides a theoretical and experimental basis for research on the occurrence and prevention of paternal diseases and the promotion of “PoHaD” theory.

## 4. Materials and Methods

### 4.1. Reagents

Nicotine (CAS No. 54-11-5) and caffeine (CAS No. C0750) were purchased from Sigma-Aldrich (St. Louis, MO, USA). Ethanol was obtained from Zhen Xin Co., Ltd. (Shanghai, China). The glucose colorimetric detection kit was purchased from Shanghai Rongsheng Biotech Co., Ltd. (Shanghai, China). The rat insulin enzyme linked immuno sorbent assay (ELISA) kit was provided by Mercodia (Uppsala, Sweden). The rat testosterone enzyme ELISA kit was provided by North Institute of Biological Technology (Beijing, China). The corticosterone (CORT) assay kit was purchased from Sangon Biotech Co., Ltd. (Shanghai, China). Total cholesterol (T-CHO), triglyceride (TG), low-density lipoprotein cholesterol (LDL-c), and high-density lipoprotein cholesterol (HDL-c) commercial kits were bought from Jiancheng Bioengineering Institute (Nanjing, China). TRIzol reagent was obtained from Thermo Fisher Scientific (Waltham, MA, USA). Reverse transcription and real-time quantitative polymerase chain reaction (RT-qPCR) kits were purchased from Takara Biotechnology (Dalian, China). Fetal bovine serum (FBS) was purchased from Gibco (Grand Island, NY, USA). All other reagents were of analytical grade.

### 4.2. Meta Analysis

Source and analysis of population research data: literature retrieval, comprehensively covers all cases of control studies and cohort studies. To identify the evidence for the relationship between paternal exposure to nicotine, ethanol, and caffeine and adverse pregnancy outcomes (stillbirth, spontaneous abortion, premature birth, low birth weight, and intrauterine retarded), we searched MEDLINE and the EMBASE database to find all studies in any language as of 11 December 2020 and retrieved a total of 764 articles related to this research. We filtered titles and abstracts to remove publications that were obviously irrelevant, such as editorials and individual case studies. The process of identifying relevant articles and extracting data was independently carried out by 6 members of the review team, and a total of 11 studies were included in the meta-analysis. Due to different experimental studies with different standards, the results were analyzed using the random effects model, and the corresponding 95% confidence interval (CI) did not cross the zero line, indicating that it was statistically significant.

Retrieval type: ((Male) OR (paternal) OR (father)) AND (((alcohol intake) OR (alcohol consumption) OR (alcohol drinking)) OR ((Caffeine intake) OR (Caffeine consumption) OR (coffee drinking)) OR ((Nicotine intake) OR (Nicotine consumption) OR (tobacco smoking))) AND ((spontaneous abortion) OR (miscarriage) OR (stillbirth) OR (preterm birth) OR (low birth weight) OR (small for gestational age infants) OR (Intrauterine Growth Retardation) OR (Growth Retardation, Intrauterine) OR (Intrauterine Growth Restriction) OR (Fetal Growth Restriction)).

### 4.3. Animals and Treatments

Specific-pathogen-free (SPF) male Wistar rats (240–270 g, 6 weeks postnatal) (certification no. 42000600002258, license No. SCXK (E) 2018-2020) were obtained from the Experimental Center of Hubei Medical Scientific Academy. Animal experiments were performed in the Animal Experimental Center of Wuhan University (Wuhan, China), which was accredited by the Association for Assessment and Accreditation of Laboratory Animal Care International (AAALAC International). The Animal Experiment Ethics Committee of Wuhan University Medical College approved the program (permit number: 201709). All animal testing procedures were performed following the guidelines of the Chinese Animal Welfare Committee on the use of experimental animals.

All rats were fed under standard conditions (room temperature: 18–22 °C; humidity: 40–60%; light cycle: 12 h light–dark cycle) for two weeks and divided into control (subcutaneously injected with normal saline daily, *n* = 15) and paternal mixed exposure (PME) groups (subcutaneously injected with nicotine (0.1 mg/kg/d), ethanol (0.5 g/kg/d), and caffeine (7.5 mg/kg/d) for 8 weeks, *n* = 15) [19,80,81]. The body weight was recorded once a week during the administration. In addition, serum samples were obtained from the orbital plexus before, 4 weeks after, and 8 weeks after administration for subsequent analysis.

After 8 weeks of administration, the male Wistar rats were mated with normal female Wistar rats in a ratio of 1:2 at 6 p.m. (each male rat was mated successfully only once). The vaginal smear was examined the next morning and the day when the sperm was observed was regarded as the gestational day (GD) 0. The pregnant female rats were picked up and kept separately. This was continued to ensure that there were at least 12 pregnant rats in each group. On GD20, pregnant rats were anesthetized with 3% isoflurane and the status of each litter (including the number of live, stillborn, and absorbed fetuses, etc.) was recorded in detail to observe the pregnancy outcomes. In addition, the serum samples of each litter of female and male fetuses were combined into one sample for subsequent analysis of serum-series-related indicators. In addition, the fetal tissues (hippocampus, adrenal, liver, ossification, testes, and ovary) were immediately frozen in liquid nitrogen and preserved at −80 °C for further analysis. Moreover, all tissues were fixed in 4% paraformaldehyde solution overnight. After dehydration, tissues were embedded within paraffin for further analysis.

After drug administration and mating, the male Wistar rats were also anesthetized with 3% isoflurane and killed. Blood was taken from the carotid artery to meet the serum index detections. In addition, the testes and adrenal glands were frozen in liquid nitrogen and placed at −80 °C for follow-up testing. The right testis of five paternal rats were randomly selected from the control group and the PME group and fixed with 4% paraformaldehyde to meet the following morphological detection. At the same time, the left caudal epididymis was obtained, the tail of epididymis was washed in culture medium preheated at 37 °C PBS and then put into a six-well plate with 4 mL PBS. The tail of the epididymis was cut into pieces and incubated at 37 °C for 2–3 min. The sperm suspension was absorbed, mixed, and diluted, and the diluted liquid was then dropped into the count plate. The sperm count and motility video detection were carried out by light microscope. In addition, the diluent was added to 4% paraformaldehyde and fixed for sperm morphological detection. All related detections were performed under the guidance of manufacturer’s instructions.

### 4.4. Cell Culture, Treatment, and Flow Cytometry

Mouse spermatogonia GC-1 cells were purchased from the Cell Bank of the Chinese Academy of Sciences (Shanghai, China) and cultured in DMEM with 10% FBS and penicillin–streptomycin (1 mM). GC-1 cells were treated with different concentrations of corticosterone (300, 600, or 1200 nM) for 24 h and harvested for further investigation. The cell cycle was analyzed via flow cytometry using a cell cycle analysis kit (Beyotime, Shanghai, China) according to the manufacturer’s instructions.

### 4.5. Hematoxylin-Eosin (H&E) Staining

The right testes were fixed in 4% paraformaldehyde overnight and processed with the paraffin sectioning technique. Sections (5 μm) were rehydrated and stained with H&E solution and then observed and photographed with an Olympus AH-2 light microscope (Olympus, Tokyo, Japan). Next, the examination and evaluation were conducted blindly by another researcher and five random fields for each section were observed under the microscope. Finally, we evaluated relevant examination indexes from each section (*n* = 5). All images were captured using an Olympus AH-2 Light Microscope (Olympus, Tokyo, Japan).

### 4.6. Immunohistochemistry and Immunofluorescence

For immunohistochemical analysis, the testis slides were heated at 60 °C for 1 h, dewaxed, rehydrated, and treated in boiling sodium citrate buffer for antigen retrieval. The immunoreactivity on reactive sections was visualized under a microscope using an SP Rabbit and Mouse HRP Kit following the manual. All images were captured using an Olympus AH-2 Light Microscope (Tokyo, Japan).

For immunofluorescent assessment, testis slides and GC-1 cell climbing slices were fixed with paraformaldehyde and permeabilized with 0.3% Triton x-100. Prepared tissue and cell climbing slices were incubated with primary antibodies overnight, then incubated with FITC-conjugated secondary antibodies. Cell nuclei were stained with DAPI (blue). Fluorescence was ultimately imaged using an Olympus IX 73 fluorescence microscope (Tokyo, Japan). All involved antibodies are listed in Supplementary Table S2.

### 4.7. Total RNA Extraction, Reverse Transcription, and RT-qPCR

Total RNA was isolated from testicular tissue using TRIzol Reagent following the manufacturer’s protocol. The total RNA was reverse transcribed using a first-strand cDNA synthesis kit. Then, RT-qPCR was performed using an SYBR Green qPCR Master Mix Kit and ABI StepOnePlus cycler (Applied Biosystems, Foster City, CA, USA). The rat primer sequences for the genes used in this study are shown in Table S2. Glyceraldehyde-3-phosphate dehydrogenase (GAPDH) was used for quantitative normalization.

### 4.8. Western Blotting

Western blot was used to determine protein expression of testicular tissues and GC-1 cells. Briefly, the extracted cytosolic proteins were separated using SDS-PAGE and then transferred onto PVDF membranes. After blocking with skim milk, the blots were incubated with primary antibodies (Table S3) and subsequently bound with corresponding secondary antibodies. The immunoblotting of target proteins was visualized with ECL reagents and captured with a chemiluminescence imager (G: BOX Chemi XRQ; Syngene, Cambridge, UK).

### 4.9. Statistical Analysis

SPSS 19 (SPSS Science Inc., Chicago, IL, USA) and Prism 7.0 (Graph Pad Software, La Jolla, CA, USA) were used for data analysis. Quantitative data were expressed as the mean ± S.E.M. A two-tailed Student’s *t*-test was used for comparisons between control and treatment groups. For studies involving more than two groups, data were evaluated with one-way analysis of variance (ANOVA) followed by Tukey’s post hoc test. *p* < 0.05 indicates statistical significance.

## Figures and Tables

**Table 1:** Correlation analyses among fetal serum corticosterone level, fetal body weight, body length, and serum IGF1 level in control and PME groups.

Fetal Indicators	CON/PME	CON/PME
	Male Serum Corticosterone	Female Serum Corticosterone
Body weight (g)	0.726 **/0.946 **	0.737 **/0.926 **
Body length (cm)	0.706 */0.836 *	0.712 */0.802 *
Serum IGF1 (ng/mL)	0.819 */0.829 *	0.737 **/0.818 **

Note: * *p* < 0.05, ** *p* < 0.01.

**Table 2:** Correlation analyses among paternal serum corticosterone/sperm mobility and fetal physical/multi-organ development in PME groups (*n* = 12).

Fetal Indicators	Paternal Serum Corticosterone ↑		Paternal Sperm Motility ↓	
	Male	Female	Male	Female
Body weight (g)	−0.543 *	−0.551 *	0.831 **	0.519 *
Body length (cm)	−0.639 **	−0.375	0.616 **	0.324
Serum corticosterone (ng/mL)	−0.593 *	−0.757 *	0. 715 **	0.575 **
Serum insulin growth factor 1 (ng/mL)	−0.602 *	−0.618 *	0.543 *	0.64 *
Serum testosterone (ng/mL)	−0.649 **	-	0.417 **	-
Serum estrogen (ng/mL)	-	−0.352	-	0.14
Serum glucose (mmol/L)	−0.286	−0.0001	0.457	0.119
Serum insulin (mmol/L)	−0.039	−0.32	0.013	0.373
Serum triglyceride (mmol/L)	0.023	0.197	−0.273	0.34
Serum total cholesterol (mmol/L)	0.061	0.454	0.107	−0.435
Serum low-density lipoprotein cholesterol (mmol/L)	−0.29	0.423	0.45	0.002
Serum high-density lipoprotein cholesterol (mmol/L)	0.073	−0.224	0.349	0.373

Note: * *p* < 0.05, ** *p* < 0.01; ↑: elevate; ↓: decrease.

## Data Availability

The data presented in this study are available on request from the corresponding author.

## Supplementary Materials

The supporting information can be downloaded at: https://www.mdpi.com/article/10.3390/ijms232315081/s1.

## Abbreviations

3β-HSD: 3β-hydroxysteroid dehydrogenase; ACC: acetyl CoA carboxylase; ACP: alkaline phosphatase; ACTH: adrenocorticotropic hormone; CORT: corticosterone; FASN: fatty acid synthase; GAPDH: glyceraldehyde 3-phosphate dehydrogenase; GD: gestational day; GR: glucocorticoid receptor; HDL-c: high-density lipoprotein cholesterol; HPA: hypothalamic-pituitary-adrenal; IGF1: insulin-like growth factor 1; IUGR: intrauterine growth retardation; LDL-c: low-density lipoprotein cholesterol; MAP2: microtubule-associated protein 2; OCN: osteocalcin; P450c11: cytochrome P450 cholesterol 11; P450scc: cytochrome P450 cholesterol side chain cleavage; PCNA: proliferating cell nuclear antigen; PME: paternal exposure to nicotine/ethanol/caffeine mixture; PSD95: post-synaptic density protein 95; RUNX2: runt-related transcription factor 2; SF1: steroidogenic factor 1; SNAP25: synaptosomal-associated protein of 25 kD; SREBP-1: sterol-regulatory element binding protein 1; StAR: steroidogenic acute regulatory protein; T-CHO: total cholesterol; TG: triglyceride.
