# Supplementary material for: Paternal Nicotine/Ethanol/Caffeine Mixed Exposure Induces Offspring Rat Dysplasia and Its Potential “GC-IGF1” Programming Mechanism
Source: Int J Mol Sci. 2022 Dec 1;23(23):15081. doi: 10.3390/ijms232315081 (PMC9737622; doi:10.3390/ijms232315081)
Supplement: Supplementary file 1 [file ijms-23-15081-s001.zip › Supplementary Materials.pdf]

# *Supplementary materials*

## Paternal nicotine/ethanol/caffeine mixed exposure induces offspring rat dysplasia and its potential "GC-IGF1" programming mechanism

### Supplementary Figures and legends

**Figure S1. Alterations in serum glucolipid metabolism after paternal exposure to nicotine/ethanol/caffeine mixtures (PME).** There were no major changes in serum glucose (A), triglyceride (TG) (B), total cholesterol (T-CHO) (C), high-density lipoprotein cholesterol (HDL-c) (D) and low-density lipoprotein cholesterol (LDL-c) (E) levels in the paternal after exposure to the nicotine/ethanol/caffeine mixture. Mean ± S.E.M., *n* = 12. CON: control.

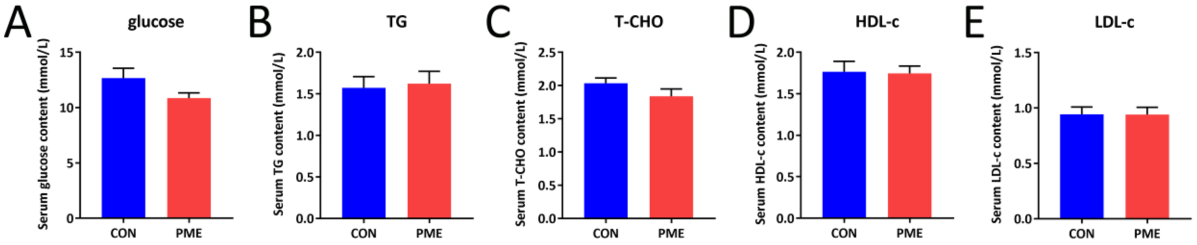


**Figure S2. Expression of proliferation markers (Ki67, PCNA) in paternal testes after exposure to nicotine/ethanol/caffeine mixtures assayed by immunohistochemistry.** Positive expression of Ki67 (A) and PCNA (B) (brown nuclei) was markedly reduced in testes of paternal exposure to the nicotine/ethanol/caffeine mixture (PME) compared to control (CON). Original magnification: 100 × (Scale bar: 100 μm); 200 × (Scale bar: 50 μm). PCNA: proliferating cell nuclear antigen.

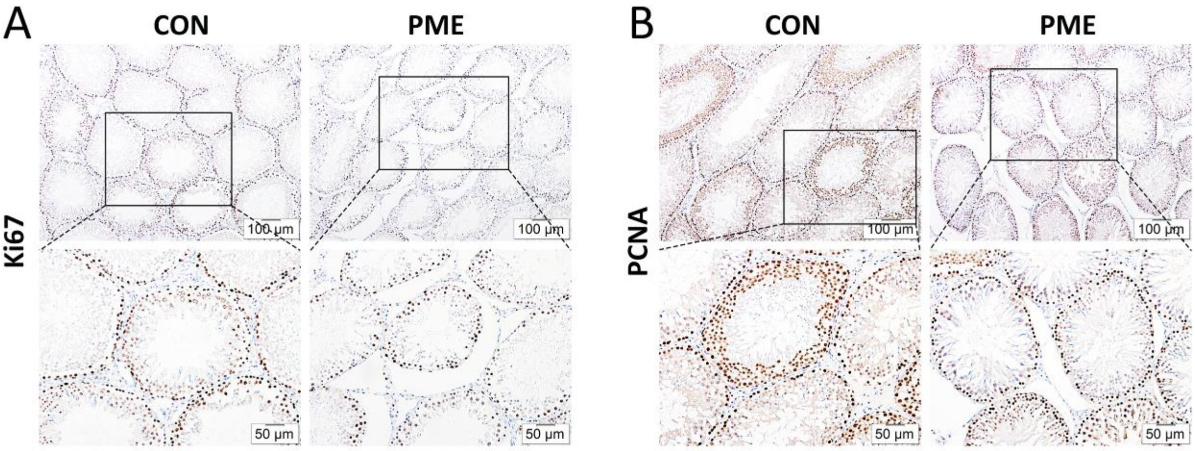

A
CON
PME
Ki67
100 μm
100 μm
50 μm
50 μm
B
CON
PME
PCNA
100 μm
100 μm
50 μm
50 μm

## Supplementary Tables

**Table S1.** Correlation analyses among paternal serum corticosterone/sperm mobility and fetal physical development/serum phenotype in control groups.

| Fetal indicators | Paternal serum corticosterone ↑ | | Paternal sperm motility ↓ | |
|---|---|---|---|---|
| | Males | Females | Males | Females |
| Body weight (g) | –0.073 | –0.175 | –0.036 | 0.13 |
| Body length (cm) | –0.443 | 0.0024 | –0.3586 | 0.094 |
| Serum corticosterone (ng/mL) | –0.668 | –0.431 | –0.6296 | –0.564 |
| Serum insulin growth factor 1 (ng/mL) | –0.058 | 0.523 | –0.2838 | –0.133 |
| Serum testosterone (ng/mL) | –0.498 | - | –0.6201 | - |
| Serum estrogen (ng/mL) | – | –0.588 | - | –0.543 |
| Serum glucose (mmol/L) | –0.346 | 0.598 | –0.3018 | 0.158 |
| Serum insulin (mmol/L) | –0.234 | –0.124 | –0.1735 | –0.406 |
| Serum triglyceride (mmol/L) | –0.341 | –0.261 | 0.3561 | 0.582 |
| Serum total cholesterol (mmol/L) | –0.362 | 0.244 | 0.3356 | –0.413 |
| Serum low-density lipoprotein-cholesterol (mmol/L) | 0.0176 | –0.026 | –0.1666 | –0.023 |
| Serum high-density lipoprotein-cholesterol (mmol/L) | 0.244 | –0.382 | –0.1068 | 0.564 |

**Table S2.** List of the primary antibodies used for Western blot (WB) analysis, immunohistochemistry (IHC), and immunofluorescence (IF) analysis.

| Protein | Antibody (and catalog number) | Application |
|---|---|---|
| GR | Rabbit polyclonal (24050-1-AP) | WB; IF * |
| Ki67 | Rabbit monoclonal (A21861) | IHC † |
| PCNA | Mouse monoclonal (2586) | IHC # |
| StAR | Rabbit monoclonal (A22166) | WB; IF † |
| GAPDH | Mouse polyclonal (AC001) | WB † |

# Provided by Cell Signaling Technology (Danvers, MA).

* Provided by Proteintech (Wuhan, China)

† Provided by ABclonal (Wuhan, China)

**Table S3.** Sequences of primers used in real-time quantitative PCR (RT-qPCR).

| **Genes** | **Forward primer** | **Reverse primer** |
|---|---|---|
| Rattus-GAPDH | GCAAGTTCAACGGCACAG | GCCAGTAGACTCCACGACA |
| Rattus-3β-HSD | CCGAAGCAGAAGCAGGTGAC | AAATGTAGTTGTGGGCGAAGC |
| Rattus-ACC | AATGAACGTGCCATCCGGTTTG | ATTCCACATTTGCATAATTGTTG |
| Rattus-ACP | GAGTCTGAGGTCTACGACACC | CCCACGGGTAATTTCTTGTCC |
| Rattus-FASN | TGCTCCCAGCTGCAGGC | CCGGTAGCTCTGGGTGTA |
| Rattus-GR | CACCCATGACCCTGTCAGTC | AAAGCCTCCCTCTGCTAACC |
| Rattus-MAP2 | TGGCTCACTTGACAATGCTCAC | TTGACCTGCTTGGCGACTGT |
| Rattus-OCN | CCGGGAGCAGTGTGAGCTTA | TAGATGCGTTTGTAGGCGGTC |
| Rattus-P450c11 | GGGCTTTGAGGCTGTCTA | CCAACCCGATCCCTTTAC |
| Rattus-P450scc | CGAGTGCCTAAGTGATAGT | AGACTGAGCCAGAAGAGC |
| Rattus-PSD95 | GTCAACACGGACACCCTAGAA | TTGATCTCCATGACCTTTTCG |
| Rattus-RUNX2 | ACCATAACAGTCTTCACAAATCCT | CAGGCGATCAGAGAACAAACTA |
| Rattus-SF1 | CTGAGGGAGACTCCTGGAAA | GTGAAATTGGTTAAGGGCATG |
| Rattus-SNAP25 | GAGCAGGTGAGCGGCATCAT | GCACGTTGGTTGGCTTCATCA |
| Rattus-SREBP-1 | GCCGTGGTGAGAAGCGCACAGCCC | CAAGACAGCAGATTTATTCAGCTTTGC |
| Rattus-StAR | GACCCGTACAGCCTACACTCG | CGAGGTTACGGCACAATCCT |
